# Sequential Semiology of Seizures and Brain Perfusion Patterns in Patients with Drug-Resistant Focal Epilepsies: A Perspective from Neural Networks

**DOI:** 10.3390/bs12040107

**Published:** 2022-04-14

**Authors:** Jorge L. Arocha Pérez, Lilia M. Morales Chacón, Karla Batista García Ramo, Lídice Galán García

**Affiliations:** 1 International Center for Neurological Restoration, 25th Ave, No 15805, Playa, Havana 11300, Cuba; jorgeluisarochaperez@gmail.com (J.L.A.P.); karlabg@infomed.sld.cu (K.B.G.R.); 2 Cuban Neurosciences Center, 25th Ave, No 15202, Playa, Havana 11300, Cuba; lidice@cneuro.edu.cu

**Keywords:** neural network, focal epilepsy, epileptogenic zone, SPECT, seizure

## Abstract

Ictal semiology and brain single-photon emission computed tomography have been performed in approaching the epileptogenic zone in drug-resistant focal epilepsies. The authors aim to describe the brain structures involved in the ictal and interictal epileptogenic network from sequential semiology and brain perfusion quantitative patterns analysis. A sequential representation of seizures was performed (n = 15). A two-level analysis (individual and global) was carried out for the analysis of brain perfusion quantification and estimating network structures from the perfusion indexes. Most of the subjects started with focal seizures without impaired consciousness, followed by staring, automatisms, language impairments and evolution to a bilateral tonic-clonic seizure (temporal lobe and posterior quadrant epilepsy). Frontal lobe epilepsy seizures continued with upper limb clonus and evolution to bilateral tonic-clonic. The perfusion index of the epileptogenic zone ranged between 0.439–1.362 (mesial and lateral structures), 0.826–1.266 in dorsolateral frontal structures and 0.678–1.507 in the occipital gyrus. The interictal epileptogenic network proposed involved the brainstem and other subcortical structures. For the ictal state, it included the rectus gyrus, putamen and cuneus. The proposed methodology provides information about the brain structures in the neural networks in patients with drug-resistant focal epilepsies.

## 1. Introduction

Epilepsy is a neural network disease [1]. It is considered a brain disease characterized by the presence of seizures, triggered by abnormal electrical discharges, which have varied clinical manifestations, multifactorial etiology and are associated with paraclinical disorders [2].

The disease can be controlled with antiepileptic drugs, but 20–30% of seizures persist or reappear, despite correct treatment [2,3,4,5,6,7,8]. According to the International League Against Epilepsy, drug-resistant epilepsy is the failure to control seizures with two antiepileptic drugs, well-tolerated, adequately selected and dosed, in mono or polytherapy [5,9].

Temporal lobe epilepsy is the most common type of focal epilepsy [7,10,11,12]. It has been conceptualized as a neural network disease that can involve brain regions beyond the mesial temporal lobe [13,14]. Frontal lobe epilepsy is the second one [15], commonly produced by Cortical Developmental Disorders [7,10,11].

Neuroimages show evidence that supports this behavior as a network in drug-resistant focal epilepsies [13,15,16,17,18]. Functional Magnetic Resonance Imaging, Positron Emission Tomography and Single-Photon Emission Computed Tomography (SPECT) are the most widely used tools. SPECT has been related to the estimation of the epileptogenic zone and the ictal onset zone.

Semiological analysis of temporal lobe seizures has suggested the activation of certain brain regions and their spread to adjacent areas [19]. Other methodologies have been reported for ictal semiology studies [20], such as cluster analysis, especially in temporal lobe epilepsy and frontal lobe epilepsy.

Brain SPECT allows the evaluation of the cortical and subcortical structures involved in drug-resistant focal epilepsies of the temporal lobe and extratemporal epilepsies. So far, it has only been performed as a qualitative method of approaching the epileptogenic zone. There has been no research in which SPECT was used to perform a quantitative analysis of the structures of the epileptogenic zone and other epileptogenic networks beyond it.

This research proposes novel aspects related to the sequential analysis of ictal semiology and the approach of the epileptogenic zone through its quantitative analysis. Perfusion indexes have been established for the brain structures in the epileptogenic zone. Furthermore, there were proposed epileptogenic networks beyond the epileptogenic zone and ictal onset zone in temporal lobe epilepsy and extratemporal epilepsies.

## 2. Materials and Methods

### 2.1. Study Setting

A monocentric, observational, cross-sectional, retrospective and prospective study was carried out in patients with drug-resistant focal epilepsies of the temporal lobe and extratemporal epilepsy at the International Center for Neurological Restoration (CIREN, from Spanish) within the epilepsy surgery program during January 2016–December 2019.

### 2.2. Study Population

Patients with drug-resistant focal epilepsies were referred from all regions of the country, with a mandatory drug-resistant condition [5,9]. Family and patient consent was received in all cases. Patients were selected from the brain SPECT database of the epilepsy surgery program, with a diagnosis of drug-resistant focal epilepsy of the temporal lobe and extratemporal epilepsy (frontal lobe epilepsy and posterior quadrant epilepsy).

### 2.3. Methodology

**Figure 1:**
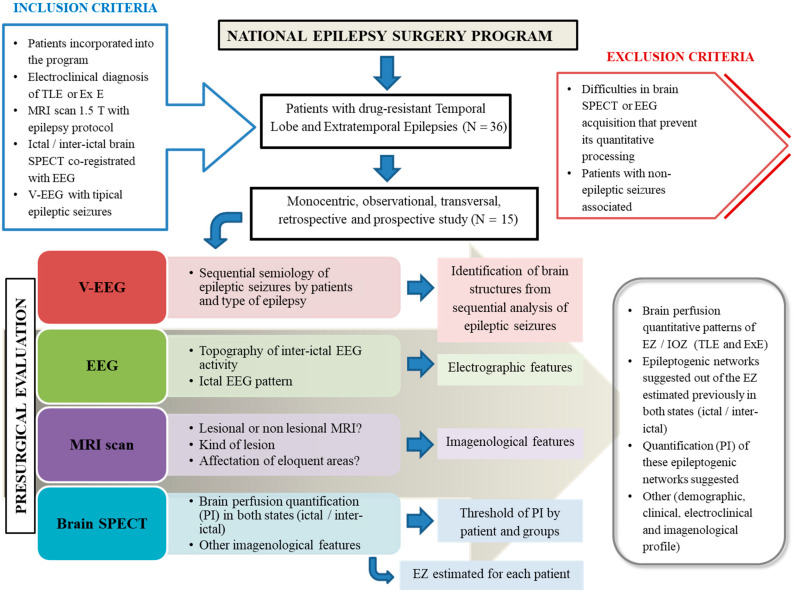
General methodological setting of this study. TLE (temporal lobe epilepsy), Ex E (extratemporal epilepsy), MRI (Magnetic Resonance Imaging), SPECT (Single-Photon Emission Computed Tomography), EEG (electroencephalography), V-EEG (Video-Electroencephalography), EZ (epileptogenic zone), IOZ (ictal onset zone).

The flowchart shows the overall setting and methodological topics (Figure 1). Subjects were evaluated according to the pre-surgical evaluation protocol approved in CIREN. It included a full medical history, complete general and neurological physical examination, as well as:

(a) Prolonged Video-Electroencephalography monitoring with scalp electrodes and additional electrodes considering the epileptogenic zone presumed;
(b) Magnetic Resonance Imaging scans with a 1.5 T scanner (Siemens Magnetom Symphony) using an epilepsy protocol [10];
(c) Interictal and ictal Electroencephalography (Micromed Software System plus Evolution and MEDICID V Amplifier System. Neuronic, Cuba) following the International System 10–20 with additional extracranial electrodes. The topography of interictal electroencephalography activity was determined, as well as the ictal electroencephalography pattern according to the different moments of the electrographic seizure;
(d) SPECT: A brain perfusion SPECT was carried out in all subjects using a double-headed gamma camera (SMV DST-XLi, Buc Cedex, France) equipped with a fan-beam collimator. Ictal SPECT was performed on just 9 subjects (2 patients with temporal lobe epilepsy, 5 patients with frontal lobe epilepsy and 2 patients with posterior quadrant epilepsy). This occurred because the ictal SPECT could not be conducted, was useless for diagnosis, and it was impossible to achieve adequate quantitative processing. In the case of interictal SPECT, all patients were studied. In both studies, the subject remained monitored by electroencephalography during the intravenous radiopharmaceutical delivery (99 mTc-ethylene-cysteine dimer). For ictal SPECT, the radiopharmaceutical was injected when electroencephalographic seizure onset was identified. The authors took into account the fact that there is a pharmacokinetic arm-brain circulation time, estimated at approximately 15–30 s for extratemporal epilepsies and temporal lobe epilepsies, respectively, which is relevant, especially in ictal SPECT. For interictal SPECT, a radiopharmaceutical was delivered with the patient lying down in a seizure-free period more than 24 h and 35 min after the radiopharmaceutical administration.

#### 2.3.1. Analysis and Processing of Information

##### Analysis of Ictal Semiology Sequences

Videos of typical seizures with adequate audio and image quality were chosen. Each sign or symptom was recorded during seizures from the beginning to end, noting the onset time of each semiological element sequentially. The glossary proposed by Dal Cól et al. [21] was taken as a reference. Subsequently, a flowchart of seizures per patient was drawn up and grouped into temporal lobe epilepsy, frontal lobe epilepsy and posterior quadrant epilepsy.

##### Quantification of Cerebral Blood Flow by SPECT

A combination of structural and functional information was required to facilitate the location of brain regions. The methodology was based on three main steps: spatial pre-processing, partial volume correction and calculation of perfusion indexes (PI). In the pre-processing, the extraction of brain and segmentation of Magnetic Resonance Imaging were carried out. The maps of gray matter, white matter and cerebrospinal fluid were obtained using the FSL program (FMRIB Software Library v5.0) [22]. The definition of regions of interest was carried out using an anatomical atlas [23]. The atlas was co-registered with the brain SPECT image. The Multi-Target Correction method was employed to correct the partial volume [24]. The perfusion index was calculated by dividing the mean activity (counts per voxel) in a given region by the mean activity of the remaining gray matter.

#### 2.3.2. Statistics Analysis

The data obtained were recorded in a STATISTICA software database (version 10, www.statsoft.com (accessed on 1 June 2019) Tulsa, OK, USA). Indicators were summarized with descriptive statistics for each variable comprising the mean, median, and standard deviations for continuous variables and frequencies for categorical ones. The Kruskal–Wallis test was applied to determine statistically significant differences between the three groups of subjects in connection with the age at the time of evaluation, number of epileptic seizures and radiopharmaceutical injection times (ictal SPECT). Additionally, Spearman’s test was applied to determine significant correlations between the radiopharmaceutical injection time and number of hyperperfused brain structures. Statistical significance was set at *p* < 0.05.

A two-level analysis was applied for studying the quantification and estimation of network structures from the perfusion indexes. The first level consisted of analysis within each subject that allowed:

- To describe the individual behavior of the distribution by subject;
- To calculate the location and dispersion parameters;
- To compare the values obtained in ictal vs non-ictal through a *t*-Student’s test;
- To determine a threshold value for discriminating both behaviors (2 discrimination methods: traditional and classification trees Breimen et al., 1984).

In the second level of analysis, the behavior of the extracted parameters was determined by calculating the location and dispersion for the threshold (mean, median, minimum and maximum values, standard deviation, 5% percentile, 95% percentile, threshold and maximum quartile).

The perfusion index threshold was taken as the individual perfusion cut-off point for interictal and ictal SPECT. From this value, the structures showing a perfusion index equal to or greater (ictal SPECT) and equal to or less (interictal SPECT) than the individual cut-off point (ipsilateral and contralateral to the epileptogenic zone) were identified. Generalities were established and such structures were considered as part of the epileptogenic network in each state for temporal lobe epilepsy and extratemporal epilepsy.

#### 2.3.3. Ethical Considerations

The procedures performed followed the rules of the Declaration of Helsinki for human research from 1975. This study was approved by the scientific and ethical committee of the International Center for Neurological Restoration (CIREN 45/2020).

## 3. Results

### 3.1. Demographic, Electroclinical and Imagenological Profile

Fifteen patients were included (Table 1). Their mean age was 24.2 ± 6.71 years (30 ± 6.29 years for temporal lobe epilepsy, 21.3 ± 5.85 years for frontal lobe epilepsy and 24 ± 5.56 years for posterior quadrant epilepsy). No statistically significant differences were found between the three groups of patients in connection with age at the time of evaluation (*p* = 0.088) and seizure duration (*p* = 0.603, Kruskal–Wallis test).

Past medical history was heterogeneous, with perinatal hypoxia being the most frequent background (N = 2). Most of the subjects (N = 10; 66%) received polytherapy. Lamotrigine, Carbamazepine and Clobazam were the most frequent prescriptions.

Table 2 shows nine patients (60%) with right laterality of the epileptogenic zone, without statistical significance between the three groups of subjects (Chi-square, *p* = 0.240848). The focal topography of the interictal electroencephalographic activity was the most frequent (8/15), followed by the regional topography (6/15), without statistical significance between the three groups (Chi-square, *p* = 0.886933).

A total of 248 seizures were analyzed in the Video Electroencephalography unit (43 in patients with temporal lobe epilepsy, 176 in frontal lobe epilepsy and 29 in posterior quadrant epilepsy). Note that the highest number of seizures took place in the wakefulness state. No statistical significances were found according to the total number of seizures (*p* = 0.983), number of seizures in wakefulness (*p* = 0.760) or during sleep (*p* = 0.989) (Kruskal–Wallis test).

The ictal electrographic activity onset in temporal lobe epilepsy was characterized by repetitive spikes and a rhythmic pattern in the theta frequency band (4.9–7.1 Hz). The mean durations of the electrographic seizure were 71.3 ± 26 s (temporal lobe epilepsy), 41 ± 31.5 s (frontal lobe epilepsy) and 29 ± 19 s (posterior quadrant epilepsy).

Regarding ictal SPECT, the sum of the injection time of radiopharmaceutical plus the arm–brain circulation time had a longer duration than the seizures in two subjects with frontal lobe epilepsy (patients 3 and 7). Therefore, in these subjects, the fixation of the radiopharmaceutical is already post-critical and brain perfusion patterns obtained after the post-processing of SPECT do not correspond to the ictal phase. In the other patients, the radiopharmaceutical reached the brain in 0.17–0.78 min (temporal lobe epilepsy), 0.28–0.41 min (frontal lobe epilepsy) and 0.30–0.35 min (posterior quadrant epilepsy), which occurred during the ictal phase, but certainly not in the initial phase.

The time between the electrographic and seizure onset ranged between 10–14 s (temporal lobe epilepsy), 0–18 s (frontal lobe epilepsy) and 0–34 s (posterior quadrant epilepsy). No statistical significances were obtained in the means between the groups in terms of seizure duration (Kruskal–Wallis test; *p* = 0.9529).

There was a predominance of non-lesional Magnetic Resonance Imaging (9/15), mainly in patients with frontal lobe epilepsy (75%). Overall, the most frequent lesion was cortical developmental disorder (four patients for 26.6%).

### 3.2. Sequential Semiological Analysis of Behavioral Seizures and Brain Perfusion Quantitative Patterns of Epileptogenic Zone during Ictal State

**Figure 2:**
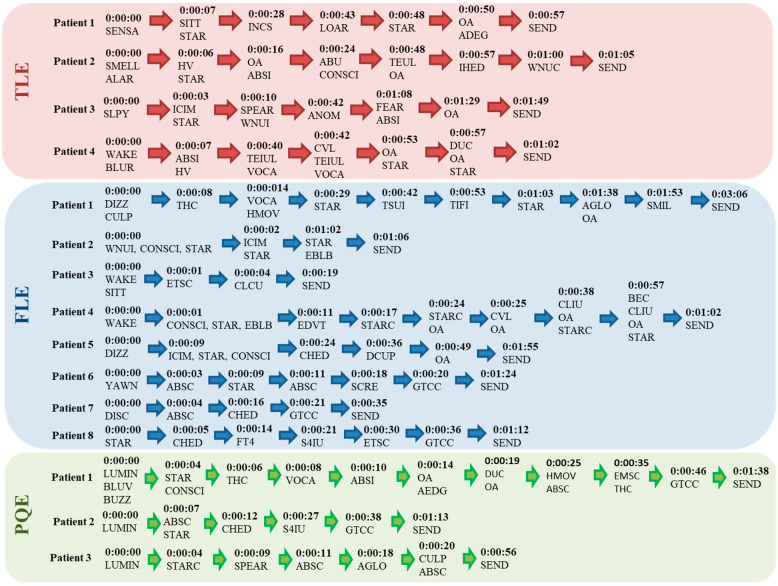
Temporal sequences of ictal behavioral patterns in patients with TLE and Ex E. TLE (temporal lobe epilepsy), FLE (frontal lobe epilepsy), PQE (posterior quadrant epilepsy), SENSA (strange sensation throughout the body), SMELL (strange smell), SLPY (sleepy), BLUR (blurred mind), SITT (sitting down), STAR (staring), INCS (incoherent speech), LOAR (looking around), OA (oral automatisms), ADEG (automatism, deglutition), ALAR (alarm), HV (hyperventilation), ABSI (automatisms, ipsilateral upper limb), ABU (automatism, bimanual), CONSCI (impaired consciousness), TEUL (tonic extension, upper limbs), IHED (ipsilateral head and eye deviation), WNUC (automatism, contralateral wiping/touching the nose), ICIM (ictal immobility), SPEAR (speech arrest), WNUI (automatism, ipsilateral wiping/touching the nose), ANOM (nominal afasia), FEAR (fear), WAKE (awakening), TEIUL (tonic extension, ipsilateral upper limb), VOCA (vocalization), CVL (cephalic version to the left), DUC (dystonia, contralateral hand), DIZZ (dizziness), LIGHT (see a light), LUMIN (luminous sparkles), BLUV (blurred vision), BUZZ (ear buzzing), THC (tonic contraction of the contralateral half face), HMOV (hyperkinesis), TSUI (tremor, ipsilateral upper limb), TIFI (tremor, ipsilateral lower limb), SMIL (smiling), ABSC (automatisms, contralateral upper limb), EMSC (elevation of the contralateral upper limb), CHED (contralateral head and eye deviation), S4IU (sign of “4” with ipsilateral arm extension), EBLB (bilateral eye blinking), GTCC (bilateral tonic-clonic), AGLO (automatism, global), STARC (contralateral staring), CULP (contralateral upper limb paresthesia), CLCU (clonus, contralateral upper limb), EDVT (eye deviation to the top), CLIU (clonus, ipsilateral upper limb), BEC (bilateral eyelid clonus), DCUP (dystonia, contralateral upper limb), YAWN (yawn), SCRE (screaming), DISC (disconnection), TF4 (tonic flexion 4 limbs), SEND (end of seizure).

Figure 2 presents the temporal sequences of seizure semiological elements in all patients. They allow proposing a temporal sequence for each epilepsy group.

In temporal lobe epilepsy, all patients started with focal seizures without impaired consciousness (“strange sensation throughout the body, strange smell, sleepy and blurred mind”) for 3–7 s. They continued with staring (7–10 s), followed by automatisms and language impairments (incoherent speech, speech arrest and vocalizations) between 28 s and 1.29 min. Subsequently, extension movements in the upper limbs appeared (40 and 48 s), finishing with automatisms. The focal dystonic posture in the contralateral hand to the epileptogenic zone (one patient) at second 57 (total duration of 1.02 min) was noteworthy.

This analysis allowed identifying the participation of structures in the epileptogenic network, initially temporal lobe mesial structures, then temporal lobe lateral regions with extension to dorsolateral prefrontal structures and Broca’s area, as well as premotor areas, belatedly. The role of ipsilateral basal ganglia in the epileptogenic zone was also found (one patient).

In temporal lobe epilepsy, the ictal SPECT was successfully performed in two patients (50%) and the radiopharmaceutical injection times ranged between 7 and 17 s (mean 12 ± 7.07 s). This allowed identifying the brain structures with the highest perfusion index at ictal onset—in order of frequency: ipsilateral parahippocampal gyrus (1.216–1.362), contralateral inferior temporal gyrus (1.072–1.179), contralateral hippocampus (1.160) and ipsilateral superior temporal gyrus (1.137).

In frontal lobe epilepsy, seizures started with staring and automatisms (50%) and focal seizures without impaired consciousness (dizziness and paresthesias) in 25% of patients. They continued with upper limb clonus (4 and 38 s). Three subjects showed an evolution to bilateral tonic-clonic seizure (between 20 and 36 s). In one subject, a tremor in the upper and lower limbs ipsilateral to the epileptogenic zone was detected before the first minute.

Semiological analysis suggested the structures related to the epileptogenic network in frontal lobe epilepsy: ipsilateral dorsolateral prefrontal region, primary motor area, basal ganglia and, finally, the entire cerebral cortex. Additionally, early injection times (mean 5.8 ± 3.19 s) during ictal SPECT helped us to determine both the cingulate gyrus (1.164–1.142) and inferior frontal gyrus (1.100–1.215) as part of the estimated epileptogenic zone.

In posterior quadrant epilepsy, seizures started as focal without impaired consciousness (to see a light, blurred vision and ear buzzing) in all patients, lasting 4–7 s. Then, the seizure continued with staring (first 6–12 s) and automatisms (first 10 s), with a subsequent evolution to a bilateral tonic-clonic seizure (n = 2) from 38 and 46 s. Note the presence of contralateral dystonic postures to the epileptogenic zone (one patient) at 19 s.

Behavioral seizures showed strong participation of the occipital lobe and lateral temporal lobe, as well as the primary auditory area initially in posterior quadrant epilepsy. Later, extension to the ipsilateral basal ganglia (one patient) and mesial temporal lobe occurred, ending on the entire cerebral cortex (two patients), parietal lobe and prefrontal regions.

The analysis of ictal perfusion patterns in posterior quadrant epilepsy (mean injection time of 4.5 ± 2.12 s) let us identify the ipsilateral superior occipital gyrus (1.507) and ipsilateral fusiform gyrus (1.393) as part of the epileptogenic zone.

No significant correlations were found between the radiopharmaceutical injection times and number of structures with perfusion index values above the threshold calculated during the ictal study (Spearman *p* = 0.590). The minimum and maximum perfusion indexes were similar in the different types of epilepsies, which do not depend on the epilepsy itself, but rather on the state (interictal or ictal) (Chi-Square test, *p* = 0.8747).

### 3.3. Brain Perfusion Quantitative Patterns of Epileptogenic Zone during Interictal State

The minimum interictal cerebral perfusion index of the epileptogenic zone structures ranged between 0.439–0.678. The maximum perfusion index was between 1.176–1.153.

For temporal lobe epilepsy, structures showed perfusion index values below the threshold—in order of frequency: ipsilateral and contralateral amygdala, ipsilateral hippocampus, and the ipsilateral and contralateral inferior temporal gyrus. In frontal lobe epilepsy, these structures were the ipsilateral and contralateral superior frontal gyrus, middle frontal gyrus (both) and the ipsilateral inferior frontal gyrus. In posterior quadrant epilepsy, these areas were the ipsilateral superior occipital gyrus, ipsilateral middle occipital gyrus, and bilateral inferior occipital gyrus.

### 3.4. Analysis of Interictal and Ictal Epileptogenic Network from the Perfusion Index

#### 3.4.1. Interictal Quantitative Perfusion

The minimum perfusions of all cerebral structures were from 0.287 to 0.638, with a mean of 0.416 ± 0.111. The media of maximum cerebral perfusion were 1.475 ± 0.347 (Table 3).

In temporal lobe epilepsy, the brain structures that integrate the epileptogenic network are the brainstem (both midbrain, ipsilateral pons and both medulla oblongata), basal ganglia and other structures of the limbic system (bilateral globus pallidus, caudate nucleus, entorhinal cortex, substantia nigra and red nucleus). The bilateral thalamus, both medial orbitofrontal gyrus and contralateral inferior occipital gyrus were also involved.

The authors found, in frontal lobe epilepsy, the following structures inside the epileptogenic network: brainstem (bilateral medulla oblongata and midbrain) and limbic system (both caudate nuclei, bilateral substantia nigra and red nucleus). Moreover, the ipsilateral thalamus, bilateral entorhinal cortex, ipsilateral hippocampus, contralateral amygdala, ipsilateral medial temporal gyrus, and bilateral fusiform gyrus, as well as the contralateral lateral orbitofrontal gyrus, ipsilateral angular gyrus, both postcentral gyrus, bilateral inferior occipital gyrus and contralateral medial occipital gyrus were involved. This network was rather extensive compared with the network described for temporal lobe epilepsy.

In posterior quadrant epilepsy, the following cerebral structures were found: medulla oblongata, midbrain, globus pallidus, substantia nigra, red nucleus, thalamus and medial orbitofrontal gyrus (all bilateral). It also included the contralateral entorhinal cortex, ipsilateral superior frontal gyrus, contralateral inferior and medial temporal gyrus.

#### 3.4.2. Ictal Quantitative Perfusion

The minimum perfusion of all brain structures (ictal state) ranged between 0.362–0.720 (mean of 0.422 ± 0.170). The mean of the maximum perfusion index was 1.464 ± 0.196 (Table 4). In all subjects, the ictal perfusion indexes of brain structures were obtained successfully; in just two patients (patients 3 and 7 with frontal lobe epilepsy), these perfusion indexes correspond to the postictal phase.

For temporal lobe epilepsy, the structures involved in the epileptogenic network (regardless of the estimated epileptogenic zone) were the ipsilateral cuneus and subcortical structures, such as the contralateral red nucleus and contralateral putamen. In frontal lobe epilepsy, the structures in this group were: ipsilateral cuneus, bilateral rectus gyrus, ipsilateral insula and bilateral putamen. Additionally, the ipsilateral inferior frontal gyrus, contralateral cingulate gyrus, ipsilateral parahippocampal gyrus, ipsilateral precuneus, ipsilateral superior occipital gyrus, contralateral cerebellum, bilateral thalamus, globus pallus and pons were identified. In the case of posterior quadrant epilepsy the epileptogenic network was also integrated with the contralateral rectus gyrus, bilateral putamen, bilateral pons and the ipsilateral parahippocampal gyrus.

#### 3.4.3. Brain Structures Proposed as Part of the Epileptogenic Network Regardless of the Type of Focal Epilepsy

There were defined common structures within the interictal epileptogenic network (determined by perfusion index) in all groups of drug-resistant focal epilepsies: bilateral brainstem, basal ganglia and other bilateral structures of the limbic system, bilateral thalamus, entorhinal cortex and frontobasal regions. The mesial and lateral temporal lobes and part of the occipital lobes were included.

## 4. Discussion

### 4.1. Demographic, Clinical and Imagenological Data

There was a predominance of males belonging to the frontal lobe epilepsy group, which matches with the literature [7,20,25,26,27]. The most frequent past medical history differed from those reported by Ortiz Giraldo [26] and Bertti [20], who declared febrile seizures, traumatic brain injury and prenatal history. Polytherapy supported the drug-resistant condition of patients being a risk factor for sudden unexpected death in epilepsy (SUDEP). Englot et al. [28] obtained similar results.

Morales Chacón et al. [11] described that the proportion of patients with non-lesional Magnetic Resonance Imaging in epilepsy surgery cohorts varies from 16% to 47%, being more frequent in extratemporal epilepsies than in temporal lobe epilepsies. Toledano et al. [7] reported non-lesional Magnetic Resonance Imaging in 46% of the subjects and focal cortical dysplasia, such as the type of Cortical Developmental Disorders, as the most frequent in lesional cases [27].

### 4.2. Sequential Semiology of Seizures

Temporal sequences allowed a different approach to the brain structures involved in the ictal onset and spreading of seizures. The novel contribution was this sequential analysis methodology in terms of epileptogenic networks. Most of the investigations were carried out in patients with temporal lobe epilepsy, frontal lobe epilepsy and posterior quadrant epilepsy, but almost none with the methodology proposed.

Bertti and collaborators [20] studied the ictal sequences by applying neuroethology and graph analysis. They reported focal seizures without impaired consciousness in temporal lobe epilepsy (mainly with epigastric symptoms), lateralized dystonia, impaired consciousness and language during the ictal and postictal periods and the evolution to bilateral tonic-clonic seizures. In frontal lobe epilepsy, they described short and frequent seizures, with motor manifestations, oculocephalic version, language impairments and a rapid postictal recovery.

The behavioral sequences proposed match partially with Bertti’s research [20], specifically with the beginning and end of seizures with automatisms; however, they did not observe focal tonic postures. According to Dal Cól [29], dystonias are frequent semiological elements in temporal lobe epilepsy, but our results do not support this. This could be caused by the small number of evaluated subjects.

Bertti et al. [20] analyzed the sequences of behavioral patterns individually in frontal lobe epilepsy. They reported axial contractions, looking around and screaming with trunk automatisms during the ictal onset, which are far from our results. These differences can be attributed to the extension of the frontal lobe.

### 4.3. Brain Perfusion Quantitative Patterns of the Epileptogenic Zone

There are no similar studies that have used the quantification of brain perfusion from perfusion indexes. The reports emanate from qualitative analysis. No relevant information was found about ictal or interictal perfusion patterns in patients with posterior quadrant epilepsy.

In temporal lobe epilepsy, the hypoperfused structures (interictal study) were the mesial and lateral temporal lobes. Hogan et al. [29] reported more prominent involvement in the midline of the brain and cerebellar hemispheres, bilaterally. Zhao and collaborators [30] explored temporal lobe epilepsy, and the hypoperfused structures were the anterior insula and orbitofrontal cortex. However, our results showed similarities with those found by Cleeren [31], especially in structures such as the hippocampus and amygdala.

A study with PET [30] performed in extratemporal epilepsy revealed significant hypometabolism, mainly in the ipsilateral orbitofrontal cortex, the anterior cingulate cortex and the anterior insula in patients with frontal lobe epilepsy. The results of the current investigation mostly contrast with Zhao’s report, probably because of frontal lobe extension.

Radiopharmaceutical delivery as soon as possible in ictal SPECT (first 15 s) after the seizure onset is crucial for accurate localization of the epileptogenic zone. In general, the radiopharmaceutical could be administered in the first 2–10 s (88%), guaranteeing an adequate acquisition of ictal SPECT and post-processing of brain perfusion quantification [32].

Cleeren [31] and Wichert [33] determined the hyperperfusion of the ipsilateral temporal lobe in most of the subjects with TLE. Santos et al. [5] described a hyperperfusion pattern in the left frontal lobe in one subject with frontal lobe epilepsy obtained by quantitative SPECT analysis.

In this study, the brain perfusion patterns in most subjects obtained from ictal SPECT showed hyperperfusion in brain structures related to an epileptogenic zone and also the spreading of the seizure. This happened due to the normal delay of radiopharmaceutical arm-brain circulation after its injection. Additionally, there were just two patients (frontal lobe epilepsy) in whom cerebral uptake of the radiopharmaceutical occurred in the postictal period, so the authors did not include them in the ictal SPECT analysis.

### 4.4. Analysis of Interictal and Ictal Epileptogenic Network

Englot and collaborators [13] observed a reduction in the functional and structural connectivity of the ascending reticular activator system between it and the neocortex. This study revealed the underestimated role of brainstem neural networks in epilepsy, especially in temporal lobe epilepsy. Evidence of brain abnormalities in temporal lobe epilepsy using functional neuroimaging [1] has included dysfunction in limbic networks and other specific brain networks, as well as global changes in network topography; aspects that coincide with our results.

There is evidence from a neuropsychological viewpoint [5] that supports the involvement of the temporal lobe in frontal lobe epilepsy. Blanco Beregaña [34] reported that the pediatric population with frontal lobe epilepsy has extensive cognitive and behavioral disorders. This fact responds to the disruption of neural networks between the frontal and temporal lobes.

There were brain structures that showed broad participation within the interictal epileptogenic network, regardless of the type of epilepsy. Bilateral brainstem structures are associated with a high risk of SUDEP [13,35,36,37].

The ENIGMA study (Enhancing Neuro Imaging Genetics through Meta-Analysis) [38] was a pioneer in the study of brain connectivity in epilepsies. The authors described the same volumetric changes in patients with mesial temporal lobe epilepsy.

According to the ictal study, Liu et al. [39] observed hyperperfusion in the ipsilateral rectus gyrus and ipsilateral insula in orbitofrontal epilepsy. Despite the difficulty of Newton et al. [40] in estimating the epileptogenic zone in extratemporal epilepsy with SPECT, we achieved it successfully.

## 5. Conclusions

Sequential behavioral representation suggested the connectivity of cortical and subcortical brain structures. It was possible to quantify the perfusion indexes of brain structures in the epileptogenic zone. The proposed methodology permitted suggesting an interictal and ictal epileptogenic network beyond the epileptogenic zone estimated.

## Figures and Tables

**Table 1:** Demographic and clinical profile.

Epilepsy Type		Subjects	Seizure Onset Y Mean/SD		Age at Evaluation Y Mean/SD		Sex	Past Medical History	Epilepsy Duration (Y)	Antiepileptic Drugs
TLE		Patient 1	34	12.2 ± 14.8	35	30.2 ± 6.29	F	Migraine	1	LTG
		Patient 2	6		21		F	No	15	CBZ
		Patient 3	8		32		F	Depression	24	LTG
		Patient 4	0.8		33		F	No	33	LTG, CBZ
Ex E	FLE	Patient 1	6	7.37 ± 6.28	21	21.3 ± 5.85	M	BA	15	CBZ
		Patient 2	4		15		M	No	11	VPA, LEV, LTG, Clobazam
		Patient 3	14		15		F	No	1	TPM, Clobazam
		Patient 4	11		31		M	PI	20	CBZ, VPA
		Patient 5	0		23		M	PH	23	LTG, Clobazam
		Patient 6	18		21		M	CF	3	CBZ, Clobazam
		Patient 7	3		17		M	No	14	CBZ, Clobazam
		Patient 8	3		28		M	AC	25	OXC, LTG, Clonazepam
	PQE	Patient 1	18	11.6 ± 5.51	29	24 ± 5.56	F	No	11	LV, LTG
		Patient 2	9		25		F	PH	16	CBZ, Clobazam
		Patient 3	8		18		M	No	10	LTG

Y (years), SD (Standard Deviation), TLE (temporal lobe epilepsy), Ex E (extratemporal epilepsy), FLE (frontal lobe epilepsy), PQE (posterior quadrant epilepsy), BA (bronchial asthma), PI (prolonged icterus), PH (perinatal hypoxia), FS (febrile seizure), BA (brain abscess), LTG (lamotrigine), CBZ (carbamazepine), VPA (valproic acid), LEV (levetiracetam), TPM (topiramate), OXC (oxcarbazepine).

**Table 2:** Electroclinical and imagenological profile.

		TLE				Ex E										
						FLE								PQE		
		Patient 1	Patient 2	Patient 3	Patient 4	Patient 1	Patient 2	Patient 3	Patient 4	Patient 5	Patient 6	Patient 7	Patient 8	Patient 1	Patient 2	Patient 3
Number of epileptic seizures	Awake	17	6	6	4	4	96	6	27	6	4	6	2	7	8	6
	Sleep	3	1	1	5	1	5	10	2	1	1	2	3	5	3	0
	Total	20	7	7	9	5	101	16	29	7	5	8	5	12	11	6
	Mean/SD	Awake (8 ± 5.9) Sleep (2.5 ± 1.9) Total (10.7 ± 6.2)				Awake (17.6 ± 30.3) Sleep (3.1 ± 2.9) Total (20.7 ± 31.1)								Awake (7 ± 1) Sleep (2.6 ± 2.5) Total (9.6 ± 3.2)		
Laterality of EZ		R	L	L	L	R	R	R	R	R	L	L	R	R	R	L
Topography of interictal EEG activity		R	R	F	F	F	M	R	R	R	R	F	R	R	F	F
EEG ictal pattern according to electrographic seizure time	<20 s	-	-	RS	RSSF	RS	RS	FRD	-	-	-	RSSF	RSSF	-	RS	RSSF
	20–59 s	-	-	RS	GLS	GLS	RS	GLS	-	-	-	NOR	GLS	-	GLS	GLS
	≥1 min	-	-	RSSF	GLS	NOR	GLS	NOR	-	-	-	NOR	NOR	-	GLS	NOR
Injection times (s) of radiopharmaceutical (ictal SPECT)		-	-	7	17	2	8	4	-	-	-	10	5	-	3	6
Duration of de electrographic seizure (s) Mean/SD	Mean/SD	-	-	97	72	95	45	16	-	-	-	14	20	-	34	8
		71.3 ± 26				41 ± 31.5								29 ± 19		
		76.8 ± 118.4 *														
Time between behavioral pattern onset and electrographic seizure onset (s)	Mean/SD	-	-	14	10	9	18	2	-	-	-	0	0	-	34	0
		8 ± 7.21				9.28 ± 12.7								11.3 ± 19.6		
		8.7 ± 11 *														
Duration of epileptic seizures (minutes) Mean/SD		0.57	1.05	1.49	1.02	3.06	1.06	0.19	1.02	1.55	1.24	0.35	1.12	1.38	1.13	0.56
		1.03 ± 0.37				1.19 ± 0.8								1.02 ± 0.42		
MRI evidence of lesion		L	NL	NL	L	NL	L	NL	NL	NL	L	NL	NL	L	L	NL
Type of lesion		HS	-	-	CNST	-	CDD	-	-	-	CDD	-	-	CDD	CDD	
Affectation of eloquent area		Y	N	N	Y	Y	Y	Y	N	N	Y	N	N	Y	N	N

* Mean and SD based on the total of patients. SD (Standard Deviation), TLE (temporal lobe epilepsy), Ex E (extratemporal epilepsy), FLE (frontal lobe epilepsy), PQE (posterior quadrant epilepsy), EZ (epileptogenic zone), MRI (Magnetic Resonance Imaging), R (right), L (left), F (focal), R (regional), M (multifocal), RS (repetitive spikes), RSSF (repetitive spikes with a specific frequency), GLS (generalized or lateral suppression), FRD (fast rapid discharge), NOR (normal electric discharge), L (lesional), NL (nonlesional), HS (hippocampal sclerosis), CNST (central nervous system tumor), CDD (cortical developmental disorder), Y (yes), N (no).

**Table 3:** Brain structures with values lower than the established threshold of Perfusion Index (interictal state).

		TLE				Ex E										
						FLE								PQE		
		Patient 1	Patient 2	Patient 3	Patient 4	Patient 1	Patient 2	Patient 3	Patient 4	Patient 5	Patient 6	Patient 7	Patient 8	Patient 1	Patient 2	Patient 3
**Threshold of PI**		0.854	0.855	0.965	0.927	0.951	0.924	0.969	0.944	0.956	0.987	0.975	0.883	0.950	0.917	0.950
**Brain** **structures**	STG i	-	-	-	-	0.942	-	-	-	0.941	-	-	-	-	-	-
	STG c	-	-	-	-	0.920	-	-	-	-	-	-	-	-	-	0.920
	MTG i	-	-	-	-	0.917	0.839	-	-	-	0.941	0.899	-	-	0.846	-
	MTG c	-	-	-	-	-	-	-	-	-	0.911	0.931	-	0.911	-	0.915
	ITG i	-	-	-	-	0.940	-	-	-	-	0.890	-	-	-	-	-
	ITG c	-	-	-	-	-	0.906	-	-	-	0.902	0.917	-	0.887	-	0.880
	A i	-	-	-	-	0.934	0.864	-	-	-	-	-	0.772	-	-	-
	A c	-	-	-	0.864	0.916	0.507	0.888	0.943	-	0.869	0.892	-	0.840	-	-
	H i	-	-	-	-	-	0.856	0.626	0.873	0.888	-	0.934	-	-	0.904	-
	H c	-	-	-	-	-	0.878	0.936	-	-	-	0.833	-	-	-	-
	PHG i	-	-	-	-	0.932	-	-	-	-	-	-	0.817	-	-	-
	PHG c	-	-	-	-	-	-	-	-	-	-	-	-	-	-	-
	CingG i	-	-	-	-	-	-	-	-	-	-	-	-	-	-	-
	CingG c	-	-	-	-	-	-	-	-	-	-	-	-	-	-	-
	SFG i	-	-	-	-	-	-	-	-	-	0.954	-	-	0.942	0.807	-
	SFG c	-	-	0.885	-	-	-	-	-	-	-	-	-	-	0.856	-
	MFG i	-	-	-	-	-	-	-	-	-	-	-	-	-	-	0.932
	MFG c	-	-	0.828	-		-	-	-	-	-	-	-	-	-	-
	IFG i	-	-	0.911	-	0.951	-	-	-	-	-	0.974	-	-	-	0.882
	IFG c	-	-	0.885	-	-	-	-	-	-	-	-	-	-	-	-
	SPG i	-	-	-	-	-	-	-	0.845	-	0.884	0.956	-	-	-	-
	SPG c	-	-	-	-	-	-	-	-	-	-	-	-	-	-	-
	PrecG i	-	-	0.854	-	-	-	-	-	-	-	0.959	-	-	-	0.919
	PrecG c	-	-	0.806	-	-	-	-	-	-	-	-	-	-	-	-
	PostG i	-	-	-	0.891	-	-	-	0.936	0.895	0.912	0.948	0.871	-	-	0.854
	PostG c	-	-	-	-	-	0.895	-	-	0.932	0.936	0.908	-	-	-	0.796
	AG i	-	-	-	-	-	-	-	0.838	0.902	0.824	0.846	-	-	-	-
	AG c	-	-	-	-	-	-	-	0.937	-	0.817	0.930	-	-	-	-
	Supram i	-	-	-	0.923	-	-	-	-	-	0.846	0.909	-	-	-	-
	Supram c	-	-	0.463	-	-	-	-	-	-	0.918	-	-	-	-	-
	Precuneus i	-	-	-	-	-	-	-	-	-	-	-	-	-	-	-
	Precuneus c	-	-	0.946	-	-	-	-	-	-	-	-	-	-	-	-
	Cuneus i	-	-	-	0.901	-	-	-	-	-	-	-	-	-	-	-
	Cuneus c	-	-	0.895	-	-	-	0.934	-	-	-	-	-	-	-	-
	LingG i	-	-	0.945	-	-	-	-	-	-	-	-	-	-	-	-
	LingG c	-	-	-	-	-	-	-	-	-	-	-	-	-	-	-
	SOG i	-	-	-	-	-	-	-	-	0.904	-	-	-	-	-	-
	SOG c	-	-	0.934	0.904	-	0.866	0.881	-	0.845	-	-	-	-	-	-
	FusifG i	-	-	-	-	0.784	-	0.868	-	0.865	0.970	-	-	-	-	-
	FusifG c	-	-	0.963	-	0.833	-	0.908	-	0.854	0.929	-	-	-	-	-
	IOG i	-	-	-	0.856	0.801	-	0.858	0.881	0.886	0.704	0.807	-	-	-	-
	IOG c	0.835	-	-	0.867	0.865	0.903	0.947	0.687	0.808	-	0.856	-	-	-	-
	MOG i	-	-	-	0.827	-	-	0.944	-	-	0.878	0.903	-	-	-	-
	MOG c	-	-	-	0.679	0.869	-	0.829	0.860	0.929	-	-	-	-	-	-
	MOFG i	-	0.832	-	0.663	0.818	-	-	-	-	-	-	-	0.912	-	0.829
	MOFG c	0.804	0.851	-	-	0.943	0.886	0.890	-	-	0.932	0.893	-	0.800	-	0.287
	LOFG i	-	-	-	0.911	0.844	-	-	-	-	0.965	0.932	-	-	-	0.764
	LOFG c	-	-	-	-	0.819	0.896	0.952	0.906	-	0.977	0.927	-	-	-	0.641
	STRAG c	-	-	0.875	-	-	-	-	-	-	-	-	-	-	-	-
	Entor i	0.791	0.741	-	0.623	0.751	-	0.855	0.909	0.877	0.756	0.856	-	-	-	0.924
	Entor c	0.727	-	0.949	0.850	0.924	-	-	0.904	0.899	0.929	-	0.801	0.785	-	0.741
	Ínsul i	-	-	-	-	-	-	-	-	-	-	-	-	-	-	-
	Ínsul c	-	-	-	-	-	-	-	-	-	-	-	-	0.937	-	-
	Cerebellum i	-	-	0.562	-	0.900	-	0.931	0.909	-	-	-	-	0.916	-	-
	Cerebellum c	-	-	-	-	0.911	-	-	-	-	-	-	-	-	-	0.946
	RedN i	0.324	0.408	-	0.677	0.410	0.316	0.416	0.761	0.708	0.758	0.855	0.392	0.341	0.768	0.484
	RedN c	0.496	0.319	-	0.857	0.303	0.314	0.695	0.898	0.672	0.787	0.961	0.406	0.528	0.639	0.383
	SNig i	0.453	0.645	-	0.579	0.565	0.488	0.553	-	-	0.574	0.586	0.700	0.614	-	0.500
	SNig c	0.543	0.616	-	0.621	0.546	0.444	0.565	0.883	-	0.815	0.923	0.458	0.595	-	0.627
	CN i	0.824	-	-	0.679	-	0.815	0.852	-	0.807	0.973	0.929	-	0.887	-	-
	CN c	0.803	0.824	-	-	0.843	0.817	0.959	-	0.908	0.968	0.934	0.879	-	0.862	-
	P c	-		-	-	-	-	-	-	-	-	-	-	0.946	-	-
	T i	0.808	0.819	-	0.692	0.833	0.848	0.805	-	0.879	-	-	0.785	0.771	-	0.825
	T c	0.769	-	0.948	0.799	0.775	0.845	0.803	-	-	-	-	0.857	0.898	-	0.752
	GP i	0.649	0.618	0.955	0.683	0.767	0.700	0.930	-	-	-	-	0.613	0.870	-	0.817
	GP c	0.653	0.607	-	0.837	0.819	0.675	0.805	-	-	-	-	0.609	0.703	-	0.849
	M i	0.651	0.728	0.942	0.813	0.671	0.689	0.815	0.828	0.801	0.806	0.945	0.756	0.862	0.902	-
	M c	0.679	0.623	-	0.812	0.575	0.584	0.783	0.859	0.769	0.901	-	0.731	0.882	0.847	0.764
	Pons i	-	-	0.845	0.921	0.844	-	0.908	-	-	0.850	-	-	-	-	-
	Pons c	0.809	-	-	-	-	0.665	0.922	-	-	0.945	-	0.869	0.893	-	-
	MO i	0.556	0.589	-	0.434	0.391	0.292	0.621	0.638	0.560	0.609	0.610	0.654	0.756	0.625	-
	MO c	0.523	0.478	-	0.538	0.358	0.461	0.562	0.753	0.718	0.413	0.622	0.648	0.624	0.474	-

TLE (temporal lobe epilepsy), Ex E (extratemporal epilepsy), FLE (frontal lobe epilepsy), PQE (posterior quadrant epilepsy), PI (perfusion index), c (contralateral), i (ipsilateral), STG (superior temporal gyrus), MTG (medial temporal gyrus), ITG (inferior temporal gyrus), A (amygdala), H (hippocampus), PHG (parahippocampal gyrus), CingG (cingulate gyrus), SFG (superior frontal gyrus), MFG (medial frontal gyrus), IFG (inferior frontal gyrus), SPG (superior parietal gyrus), PrecG (precentral gyrus), PostG (postcentral gyrus), AG (angular gyrus), Supram (supramarginal gyrus), LingG (lingual gyrus), SOG (superior occipital gyrus), FusifG (fusiform gyrus), IOG (inferior occipital gyrus), MOG (medial occipital gyrus), MOFG (medial orbitofrontal gyrus), LOFG (lateral orbitofrontal gyrus), STRAG (rectus gyrus), Entor (entorhinal area), Insul (insula), RedN (red nucleus), SNig (substantia nigra), CN (caudate nucleus), P (putamen), T (thalamus), GP (globus pallidus), M (midbrain), MO (medulla oblongata).

**Table 4:** Brain structures with values above the established threshold of the Perfusion Index (ictal state).

		TLE				Ex E										
						FLE								PQE		
		Patient 1	Patient 2	Patient 3	Patient 4	Patient 1	Patient 2	Patient 3	Patient 4	Patient 5	Patient 6	Patient 7	Patient 8	Patient 1	Patient 2	Patient 3
**Threshold of PI**		-	-	1.171	1.148	1.118	1.114	-	-	-	-	-	1.142	-	1.155	1.154
**Brain structures**	STG i	-	-	-	1.252	-	-	-	-	-	-	-	-	-	-	-
	A i	-	-	-	-	-	-	-	-	-	-	-	-	-	1.493	-
	A c	-	-	-	1.222	-	-	-	-	-	-	-	-	-	-	-
	PHG i	-	-	-	-	1.143	-	-	-	-	-	-	-	-	1.378	1.409
	PHG c	-	-	-	-	-	-	-	-	-	-	-	-	-	-	-
	CingG i	-	-	-	-	-	-	-	-	-	-	-	-	-	-	1.227
	CingG c	-	-	-	1.154	-	-	-	-	-	-	-	-	-	-	1.205
	IFG i	-	-	-	-	-	-	-	-	-	-	-	-	-	1.187	-
	IFG c	-	-	-	-	-	-	-	-	-	-	-	-	-	1.240	-
	SPG i	-	-	-	-	-	-	-	-	-	-	-	-	-	-	-
	SPG c	-	-	-	-	-	-	-	-	-	-	-	-	-	-	1.222
	PrecG i	-	-	-	-	1.126	-	-	-	-	-	-	-	-	-	-
	PostG i	-	-	1.237	-	-	-	-	-	-	-	-	-	-	-	-
	Supram c	-	-	-	-	-	-	-	-	-	-	-	-	-	-	-
	Precuneus i	-	-	-	-	1.247	-	-	-	-	-	-	-	-	-	-
	Precuneus c	-	-	-	-	-	-	-	-	-	-	-	-	-	-	-
	Cuneus i	-	-	1.188	1.162	-	-	-	-	-	-	-	1.224	-	-	-
	Cuneus c	-	-	-	1.403	-	-	-	-	-	-	-	-	-	-	-
	LingG i	-	-	-	1.172	-	-	-	-	-	-	-	-	-	-	-
	SOG i	-	-	1.249	-	1.259	-	-	-	-	-	-	-	-	1.507	-
	IOG c	-	-	-	-	1.180	-	-	-	-	-	-	-	-	-	-
	MOFG i	-	-	-	-	1.180	-	-	-	-	-	-	-	-	-	-
	MOFG c	-	-	-	-	1.236	-	-	-	-	-	-	-	-	1.847	-
	LOFG i	-	-	-	-	-	-	-	-	-	-	-	-	-	-	-
	LOFG c	-	-	-	-	1.121	-	-	-	-	-	-	-	-	1.346	-
	STRAG i	-	-	-	-	1.379	-	-	-	-	-	-	1.166	-	-	1.368
	STRAG c	-	-	-	-	1.206	-	-	-	-	-	-	-	-	1.530	1.579
	Entor i	-	-	-	-	-	-	-	-	-	-	-	1.196	-	1.191	-
	Entor c	-	-	-	1.205	-	-	-	-	-	-	-	-	-	1.445	-
	Ínsul i	-	-	-	-	1.229	-	-	-	-	-	-	1.174	-	-	1.254
	Ínsul c	-	-	-	-	-	-	-	-	-	-	-	-	-	-	1.259
	Cerebellum c	-	-	1.176	-	-	-	-	-	-	-	-	-	-	-	-
	RedN c	-	-	1.592	1.205	-	-	-	-	-	-	-	-	-	-	-
	SNig c	-	-	1.333	-	-	-	-	-	-	-	-	-	-	-	-
	CN c	-	-	1.446	-	-	-	-	-	-	-	-	-	-	-	-
	P i	-	-	-	1.409	1.236	-	-	-	-	-	-	1.382	-	1.277	1.179
	P c	-	-	1.386	1.387	1.120	-	-	-	-	-	-	1.515	-	1.236	1.244
	T i	-	-	-	-	-	-	-	-	-	-	-	1.239	-	-	-
	T c	-	-	-	1.270	-	-	-	-	-	-	-	1.210	-	-	-
	GP i	-	-	-	1.364	-	-	-	-	-	-	-	1.346	-	1.356	-
	GP c	-	-	1.567	1.325	-	-	-	-	-	-	-	1.203	-	-	-
	M c	-	-	-	1.179	-	-	-	-	-	-	-	-	-	-	-
	Pons i	-	-	-	1.204	-	-	-	-	-	-	-	1.174	-	1.240	1.262
	Pons c	-	-	-	1.262	1.132	-	-	-	-	-	-	1.533	-	1.193	1.254

TLE (temporal lobe epilepsy), Ex E (extratemporal epilepsy), FLE (frontal lobe epilepsy), PQE (posterior quadrant epilepsy), PI (perfusion index), c (contralateral), i (ipsilateral), STG (superior temporal gyrus), A (amygdala), PHG (parahippocampal gyrus), CingG (cingulate gyrus), IFG (inferior frontal gyrus), SPG (superior parietal gyrus), PrecG (precentral gyrus), Supram (supramarginal gyrus), LingG (lingual gyrus), SOG (superior occipital gyrus), IOG (inferior occipital gyrus), MOFG (medial orbitofrontal gyrus), LOFG (lateral orbitofrontal gyrus), STRAG (rectus gyrus), Entor (entorhinal area), Insul (insula), RedN (red nucleus), SNig (substantia nigra), CN (caudate nucleus), P (putamen), T (thalamus), GP (globus pallidus), M (midbrain).

## Data Availability

The data presented in this study are available on request from the corresponding author. The data are not publicly available to protect participant privacy.

## References

[1] Abbott D.F., Archer J.S., Carney P.W., Vaughan D.N., Jackson G.D. (2019). Functional Brain Mapping of Epilepsy Networks: Methods and Applications. Front. Neurosci..

[2] Bender J.E. (2018). Epilepsy, a burden health problem. Rev. Haban Cienc. Méd..

[3] Stamoulis C., Verma N., Kaulas H., Halford J., Duffy F.H., Pearl P.L., Treves S.T. (2017). The promise of subtraction ictal SPECT co-registered to MRI for improved seizure localization in pediatric epilepsies: Affecting factors and relationship to the surgical outcome. Epilepsy Res..

[4] Begueria R. (2018). Manual de Prácticas Médicas.

[5] Santos A.S., Chacón L.M., Romanidy M.U., Hernández L.P., Vázquez V.R., García-Ramo K.B. (2020). Epileptogenic zone surgery located in an eloquent area of the frontal lobe in an adolescent with epilepsy. Rev. Cubana Neurol. Neurocir..

[6] Rings T., von Wrede R., Lehnertz K. (2019). Precursors of seizures due to specific spatial-temporal modifications of evolving large-scale epileptic brain networks. Nature.

[7] Toledano R., Martínez-Álvarez R., Jiménez-Huete A., García-Morales I., Aledo-Serrano Á., Cabrera W., Rey G., Campo P., Gómez-Angulo J., Blumcke I. (2019). Estereoelectroencefalografía en la evaluación prequirúrgica de epilepsias focales refractarias: Experiencia de un centro de epilepsia. Neurología.

[8] Morales L.M., Báez M.M., Bender J.E., González J., García M.E., Lorigados L., González M.E., Díaz M.L., Estupiñán B.O., Pavón N. (2018). Drug-resistant epilepsies. Treatment in Cuba.

[9] Andalusian Epilepsy Society (2020). Clinical Practice Guideline 2020. Diagnostic and Treatment of Epilepsy.

[10] Bernasconi A., Cendes F., Theodore W.H., Gill R.S., Koepp M.J., Hogan R.E., Jackson G., Federico P., Labate A., Vaudano A.E. (2019). Recommendations for the use of structural magnetic resonance imaging in the care of patients with epilepsy: A consensus report from the International League against Epilepsy Neuroimaging Task Force. Epilepsia.

[11] Chacón L.M.M., Catasus C.A.S., Martin M.M.B., Rojas R.R., Pedre L.L., Diaz B.E. (2015). Multimodal imaging in nonlesional medically intractable focal epilepsy. Front. Biosci..

[12] Morales Chacón L.M., Garcia Maeso I., Baez Martin M.M., Bender del Busto J.E., García Navarro M.E., Quintanal Cordero N., Estupiñan Díaz B., Lorigados Pedre L., Valdés Yerena R., Gonzalez J. (2018). Long-Term Electroclinical and Employment Follow up in Temporal Lobe Epilepsy Surgery. A Cuban Comprehensive Epilepsy Surgery Program. Behav. Sci..

[13] Englot D.J., Gonzalez H.F., Reynolds B.B., Konrad P.E., Jacobs M.L., Gore J.C., Landman B.A., Morgan V.L. (2018). Relating structural and functional brainstem connectivity to disease measures in epilepsy. Neurology.

[14] Morgana V.L., Changa C., Englot D.J., Rogers B.P. (2020). Temporal lobe epilepsy alters spatio-temporal dynamics of the hippocampal functional network. NeuroImage Clin..

[15] Yang H., Ren J., Wang Q. (2018). Abnormal Brain Network in Epilepsy and Associated Comorbidites. Neuropsychiatry.

[16] Maestú F., Pereda E., del Pozo F. (2015). Conectividad funcional y anatómica en el cerebro humano. Análisis de Señales y Aplicaciones en Ciencias de la Salud.

[17] Frings L., Schulze A., Spreer J., Wagner K. (2009). Reduced interhemispheric hippocampal BOLD signal coupling related to early epilepsy onset. Seizure.

[18] Zhang Z., Lu G., Zhong Y., Tan Q., Liao W., Chen Z., Shi J., Liu Y. (2009). Impaired perceptual networks in temporal lobe epilepsy revealed by resting fMRI. J. Neurol..

[19] Bertti P., Dal-Cól M.L.C., Wichert-Ana L., Kato M., Terra V.C., de Oliveira J.A.C., Velasco T.R., Sakamoto A.C., Garcia-Cairasco N. (2010). The neurobiological substrates of behavioral manifestation during temporal lobe seizures: A neuroethological and ictal SPECT correlation study. Epilepsy Behav..

[20] Bertti P., Tejada J., Martins A.P.P., Dal-Cól M.L.C., Terra V., de Oliveira J.A.C., Velasco T.R., Sakamoto A.C., Garcia-Cairasco N. (2014). Looking for complexity in quantitative semiology of frontal and temporal lobe seizures using neuroethology and graph theory. Epilepsy Behav..

[21] Dal-Cól M., Terra V., Velasco T., Oliveira J., Sakamoto A., Garcia-Cairasco N. (2005). Neuroethology application for the study of human temporal lobe epilepsy: From basic to applied sciences. Epilepsy Behav..

[22] Jenkinson M., Beckmann C.F., Behrens T.E., Woolrich M.W., Smith S.M. (2012). FSL. NeuroImage.

[23] Oishi K., Faria A., Jiang H., Li X., Akhter K., Zhang J., Hsu J.T., Miller M.I., van Zijl P.C., Albert M. (2009). Atlas-Based Whole Brain White Matter Analysis Using Large Deformation Diffeomorphic Metric Mapping: Application to Normal Elderly and Alzheimer’s Disease Participants. NeuroImage.

[24] Erlandsson K., Wong A.T., van Heertum R., Mann J.J., Parsey R.V. (2006). An improved method for voxel-based partial volume correction in PET and SPECT. NeuroImage.

[25] Chacón L.M., González J.G., Batista S.H.B., García-Ramo K.B., Santos A., Ríos M. (2020). Electroclinical Profile and Outcomes in Extratemporal Lobe Epilepsy Surgery Based on Intraoperative Electrocorticography. Neurol Disord. Epilepsy J..

[26] Ortiz B., Naranjo L.F., Cornejo J.W., Solarte R.A. (2017). Clinical, electroencephalographic e imagenological features in adults with temporal lobe epilepsy of Epilepsy program of Antioquia University: Descriptive-retrospective study in Medellin 2008–2012. Acta Neurol. Colomb..

[27] Chacón L.M., González J.G., Castillo M.R., Batista S.B., García-Ramo K.B., Santos A.S., Cordero N.Q., Bermúdez M.Z., Fernández R.G., Díaz B.E. (2021). Surgical Outcome in Extratemporal Epilepsies Based on Multimodal Pre-Surgical Evaluation and Sequential Intraoperative Electrocorticography. Behav. Sci..

[28] Parker C.S., Clayden J.D., Cardoso M.J., Rodionov R., Duncan J.S., Scott C., Diehl B., Ourselin S. (2018). Structural and effective connectivity in focal epilepsy. NeuroImage Clin..

[29] Dal-Cól M.L.C., Bertti P., Terra-Bustamante V.C., Velasco T.R., Rodrigues M.C.A., Wichert-Ana L., Sakamoto A.C., Garcia-Cairasco N. (2007). Is dystonic posturing during temporal lobe epileptic seizures the expression of an endogenous anticonvulsant system?. Epilepsy Behav..

[30] Zhao B., Zhang C., Wang X., Wang Y., Mo J., Zheng Z., Ai L., Zhang K., Zhang J., Shao X.-Q. (2020). Orbitofrontal epilepsy: Distinct neuronal networks underlying electroclinical subtypes and surgical outcomes. J. Neurosurg..

[31] Cleeren E., Premereur E., Casteels C., Goffin K., Janssen P., Van Paesschen W. (2016). The effective connectivity of the seizure onset zone and ictal perfusion changes in amygala kindled rhesus monkeys. NeuroImage Clin..

[32] Theodore W.H. (2017). Presurgical Focus Localization in Epilepsy: PET and SPECT. Semin. Nucl. Med..

[33] Wichert-Ana L., Velasco T.R., Terra V., Araújo D., Júnior V.A., Kato M., Leite J.P., Assirati J.A., Machado H., Bastos A.C. (2001). Typical and atypical perfusion patterns in periictal SPECT of patients with unilateral temporal lobe epilepsy. Epilepsia.

[34] Blanco M. (2020). Alteraciones Neuropsicológicas en Epilepsia del Lóbulo Frontal en Niños.

[35] Allen L.A., Harper R.M., Guye M., Kumar R., Ogren J.A., Vos S.B., Ourselin S., Scott C.A., Lhatoo S.D., Lemieux L. (2019). Altered brain connectivity in sudden unexpected death in epilepsy (SUDEP) revealed using resting-state fMRI. NeuroImage Clin..

[36] Sanchez A.L., Fernández I.P., Principe A., Ley M., Rocamora R. (2019). SUDEP in Spain: First case series and epidemiological analysis. Seizure.

[37] Liu J., Peedicail J.S., Gaxiola-Valdez I., Li E., Mosher V., Wilson W., Perera T., Singh S., Teskey G.C., Federico P. (2020). Postictal brainstem hypoperfusion and risk factors for sudden unexpected death in epilepsy. Neurology.

[38] Whelan C.D., Altmann A., Botía J.A., Jahanshad N., Hibar D.P., Absil J., Alhusaini S., Alvim M.K.M., Auvinen P., Bartolini E. (2018). Structural brain abnormalities in the common epilepsies assessed in a worldwide ENIGMA study. Brain.

[39] Liu Y., Wang S., Hong B., Wang H., Lin J., Shi J., Zhao T., Bai J., Li J., Zhou W. (2020). Electroclinical features of lateral and medial orbitofrontal epilepsy: A case series. Epileptic Disord..

[40] Newton M.R., Berkovic S., Austin M.C., Rowe C.C., McKay W.J., Bladin P.F. (1995). SPECT in the localisation of extratemporal and temporal seizure foci. J. Neurol. Neurosurg. Psychiatry.

